# It’s about time: mitigating cancer-related cognitive impairments through findings from computational models of the Wisconsin Card Sorting Task

**DOI:** 10.1186/s12885-024-12545-7

**Published:** 2024-07-04

**Authors:** Darren Haywood, Frank D. Baughman, Evan Dauer, Jennifer Haywood, Susan Rossell, Nicolas H. Hart

**Affiliations:** 1 Human Performance Research Centre, INSIGHT Research Institute, Faculty of Health, University of Technology Sydney (UTS), Moore Park, Sydney, NSW 2030 Australia; 2 Department of Mental Health, St. Vincent’s Hospital Melbourne, Fitzroy, VIC Australia; 3 Department of Psychiatry, Melbourne Medical School, Dentistry and Health Sciences, University of Melbourne, Parkville, VIC Australia; 4 School of Population Health, Faculty of Health Sciences, Curtin University, Bentley, WA Australia; 5 Centre for Mental Health and Brain Sciences, Swinburne University of Technology, Hawthorn, VIC Australia; 6 Caring Futures Institute, College of Nursing and Health Sciences, Flinders University, Adelaide, SA Australia; 7 Exercise Medicine Research Institute, School of Medical and Health Sciences, Edith Cowan University, Perth, WA Australia; 8 Cancer and Palliative Care Outcomes Centre, Faculty of Health, Queensland University of Technology (QUT), Brisbane, QLD Australia; 9 Institute for Health Research, University of Notre Dame Australia, Perth, WA Australia

**Keywords:** CRCI, Cancer-related cognitive impairment, WCST, Psychopathology, Wisconsin card sorting task, Neurocognition, Supportive care, Cancer

## Abstract

**Background:**

Many cancer survivors experience cancer-related cognitive impairment (CRCI), often with significant negative consequences across various life domains. Emerging evidence suggests that allowing additional time to process information before acting may be a useful strategy for those with CRCI to mitigate some of its impacts. The Wisconsin Card Sorting Task (WCST), a measure of general cognition, has shown that for some cancer survivors, longer task completion time facilitates similar task performance outcomes to control populations concerning perseveration errors; a key performance metric of the WCST. However, assessing if this strategy may be useful, as well as determining for whom it may be useful, with regard to strengths and weaknesses among select cognitive domains, is challenging due to factors such as the problem of task impurity. Accordingly, this study provides an initial computational and experimental assessment of whether additional time to process information before acting is a useful strategy for those with CRCI.

**Methods:**

We simulated individual cognitive differences observed in humans by varying contributions of executive functioning components (updating, shifting, inhibition) to yield 48 distinct computational models of the WCST. Our main manipulation was then to provide these models with more or less time (at three levels of 20, 40 and 60 cycles) before models executed an action to sort a given card. We compared the number of perseveration errors on the WCST produced by the computational models. Additionally, we determined models that simulated the performance of cancer survivors on the WCST by comparing the number of perseveration errors produced by the models to human data.

**Results:**

Additional processing time resulted in the models producing significantly fewer perseveration errors, supporting our hypothesis. In addition, 8 unique models simulated the performance of cancer survivors on the WCST. Additional time appeared to have a positive influence on performance primarily by mitigating the impacts of severe inhibition impairments. For more severe global executive function impairments, a substantial amount of additional time was required to mitigate the impacts of the impairments. For the most severe impairments, additional time was unable to adequately mitigate the impact on performance.

**Conclusion:**

Additional processing time may be a useful strategy to rectify perseveration errors among cancer survivors with CRCI. Our findings have implications for the development of practical strategies, such as workload and deadline management in occupational settings, which may mitigate the negative effects of CRCI.

**Supplementary Information:**

The online version contains supplementary material available at 10.1186/s12885-024-12545-7.

## Introduction

Up to 75% of cancer survivors (i.e., people living with and beyond cancer) report experiencing cognitive impairment, commonly known as cancer-related cognitive impairment (CRCI) [1–3], characterised by impairments to higher-order cognitive domains including executive functioning (EF), and lower-level domains such as speed of information processing [3, 4]. Cancer-related cognitive impairment can exhibit transient and long-term effects enduring up to 20 years post-remission as well as immediately after the completion of active cancer treatment [1], and is demonstrated to exert substantial negative impacts on the daily life, relationships, occupational functioning, and social functioning of cancer survivors [4–6]. This has resulted in cognitive functioning being listed as a rehabilitation target in the World Health Organisation’s Package of Interventions for Rehabilitation (Cancer) [7]. Etiological hypotheses and emerging evidence suggest that cancer, cancer treatments, and psychosocial well-being each contribute to the development and persistence of CRCI see [3, 8–11]. Various supportive care strategies, such as cognitive training, exercise, psychotherapy, and pharmacological interventions, have been developed to target CRCI and continue to evolve [3]. However there is growing interest in developing practical strategies that might manage the effects of CRCI on activities of daily living [4, 5].

Qualitative and quantitative evidence suggests that providing additional time for individuals with CRCI to process information and make decisions may potentially alleviate some of its effects on functioning [4, 5, 12]. This has practical implications as it may suggest that, for example, occupational performance could be maintained if workload and timelines are managed appropriately to support cancer survivors when returning to work. The potential for cognitive compensation through increased processing time is exemplified by the performance of cancer survivors on the Wisconsin Card Sorting Task (WCST) [12, 13], a well-established measure of global cognitive functioning, encompassing higher-level processes including executive functioning (i.e., updating, shifting, and inhibition) as well as lower-level processes like speed of information processing [13, 14]. The task involves four ‘key cards’ and a set of 64 cards or 128 cards with various geometric shapes, colours, and numbers. The key cards remain constant, but in each trial, the participant selects a card from the set and matches it to one of the key cards based on a strategy of number, colour, or shape. Initially, participants are unaware of the necessary matching dimension and must identify it through trial-and-error, guided by experimenter feedback. After identifying the correct strategy, participants repeat it until the experimenter changes the matching dimension (usually after six or ten consecutive correct responses). The participant must then determine the new matching strategy, and the process continues for another dimension (colour, shape, or number). Performance on the WCST is assessed through indices such as the number of current sorts, categories completed, non-perseveration errors, and perseveration errors [13].

While all WCST outcome measures offer valuable insights into cognitive performance, perseveration errors (PE) – the continued use of a previously correct sorting strategy despite being informed that it is incorrect – has gained special attention across various populations [14] due to its consistent association and utility in predicting important outcomes across occupational (e.g., occupational performance [15], adaptive and agile decision making [16]), and psychopathology domains (e.g., Major Depressive Disorder [17, 18], obsessive-compulsive disorder [19, 20], anorexia nervosa [21], anxiety disorders [19, 22, 23], and schizophrenia [14, 24]). These outcomes are particularly salient within cancer survivorship e.g., [25–35], thus PE performance on the WCST is an appealing domain of assessment. However, the available literature indicates that, contrary to initial expectations, cancer survivors may not exhibit significantly more PE compared to controls. For example, Nguyen et al. [36] found no significant difference in PE between breast cancer survivors (women over 65 years of age who were at least 50 years old at the time of diagnosis, and at least 10 years post-treatment) who completed chemotherapy or local therapies, and controls. Similarly, Kesler et al. [11] demonstrated no significant differences in PE using a time-restricted computerised version of the WCST between chemotherapy naïve cancer survivors and healthy controls, despite showing significant differences for cancer survivors following chemotherapy. Kesler et al. [12] also found that, on the time-restricted task, cancer survivors who had chemotherapy still took significantly longer (313 s) than the chemotherapy naïve cancer survivors (267 s) and healthy controls (246 s) to complete the WCST. The authors suggested the similar number of PE between chemotherapy naïve cancer survivors may be functionally related to completion time as there was a significant association between completion time and fewer PE within the chemotherapy naïve cancer survivor group [12]. This supports the potential hypothesis that cancer survivors with CRCI might compensate for their cognitive impairments by allowing additional processing time. However, Kesler et al. [11] also observed that chemotherapy-treated cancer survivors made more perseveration errors (PE) than both controls and chemotherapy naïve cancer survivors, despite taking significantly more time to complete the task [12]. The authors suggested that slowing down may be a helpful strategy to compensate for cognitive impairments for the chemotherapy naïve group, but not for those exposed to chemotherapy.

Although it seems that cognitive compensation via additional processing time may be a viable strategy for *some* cancer survivors, understanding if, and for whom, this strategy may be useful is challenging due to the task impurity problem – which references that traditional methods of neurocognitive assessment are not direct, error free assessments of *singular* cognitive processes (i.e., a task designed to measure a particular cognitive process for example, inhibition, still also involves the functioning, and therefore a measurement, of other processes such as memory and attention shifting), and so understanding what cognitive impairments may be compensated for is challenging [37]. Accordingly, mitigating this issue to provide a mechanistic account of cognitive compensation via time in cancer survivors using traditional neurocognitive assessment methods alone is impossible. Computational modelling provides a means to articulate and directly test hypotheses and theories [14, 38, 39]. These approaches are particularly valuable for exploring the nuances embedded in cognitive functioning, through *direct* manipulation and assessment of *causal* influence. Therefore, computational modelling facilitates for the mitigation of the task impurity problem by allowing the researchers to *directly* manipulate the functioning of specific cognitive processes through code, rather than relying on in-direct and not-specific behavioural measures of each process. For instance, computational modelling has successfully explored how similar performances of distinct clinical subgroups on the WCST can be distinguished by modelling the underlying covert cognitive actions, such as stimulus-response learning [40–42]. Additionally, we have previously developed computational models of the WCST that simulated the performance of people with schizophrenia by directly manipulating components related to the EFs updating, shifting, and inhibition [14]. *Updating* refers to the process that oversees and modifies the contents of working memory to facilitate the accessibility of pertinent information for enhancing task performance. *Shifting* refers to the process that redirects attention away from one mental set and subsequently engages with another that is more suitable for the current task. *Inhibition* refers to the process that restrains automatic responses that are not pertinent to the successful completion of a task [43]. Each of these EFs are implicated in CRCI see [3, 44–47]. These examples illustrate that computational approaches can provide an effective platform for directly assessing whether cognitive impairments frequently experienced by cancer survivors can be compensated for by allowing additional time for information processing, while also exploring the nuances between profiles of cognitive function, time, and performance.

The objective of this study was to use computational modelling to directly examine the production of PE on the WCST as it is related to allowable card sorting time, as well as simulate cancer survivors’ production of PE on the WCST and provide an assessment of the simulation models’ characteristics. Specifically, this study aims to [1] examine if allowing the computational models additional card sorting time can compensate for cognitive impairments, as evidenced by the number of PE on the WCST; and [2] explore the profiles of cognitive impairments and the amount of allowable card sorting time of the computational models that simulate the number of PE performed by cancer survivors on the WCST. It is hypothesised the WCST models that (h1a) allowed for additional card sorting time would produce significantly fewer PE, and (h1b) after accounting for number of non-perseveration errors and categories obtained, this significant difference would be maintained. Further, we expected that by allowing additional card sorting time, the computational models that had significant cognitive impairments and that produced more PE than the target simulation groups, would perform a reduced number of PE that simulated the performance of cancer survivors.

## Methods

### Design

A between-group study design was used to compare the number of PE on the WCST produced by the computational models with differing allowable time before sorting each card in the WCST. Additionally, we compared the number of PE performed by the models to published research examining the performance of cancer survivors (both chemotherapy exposed and local treatments) on the WCST [36].

### Measures

Computational models performed the 128-card version of the WCST, comprising of two sets of 64 cards. The administration and scoring of the task followed the procedures given by Heaton et al. [13]. PE was calculated as the number of errors that were committed when the sorting strategy was maintained despite being informed that it is now incorrect. Non-perseveration errors were calculated as the number of all sorting errors that were not perseverative. Categories obtained referred to the number of categories completed each of which required 10 correct sorts of a single required sorting strategy. Correct sorts were calculated as the number of total correct car sorts [13].

### Computational Approach

Previously developed computational models of the WCST see [14] were utilised and extended upon. Computational models were developed and run in the software ‘Cognitive Objects Within a Graphical Environment’ (COGENT). COGENT [48] is a cognitive modelling platform that combines symbolic and connectionist approaches, using the Prolog programming language for constructing models of various systems. Unlike other systems, COGENT does not require a predefined modelling architecture, allowing for the development of purpose-fit models [14, 48, 49]. Its modelling environment employs a boxes-and-arrows approach, aligning with prevalent conceptual frameworks in neurocognition.

**Fig. 1:**
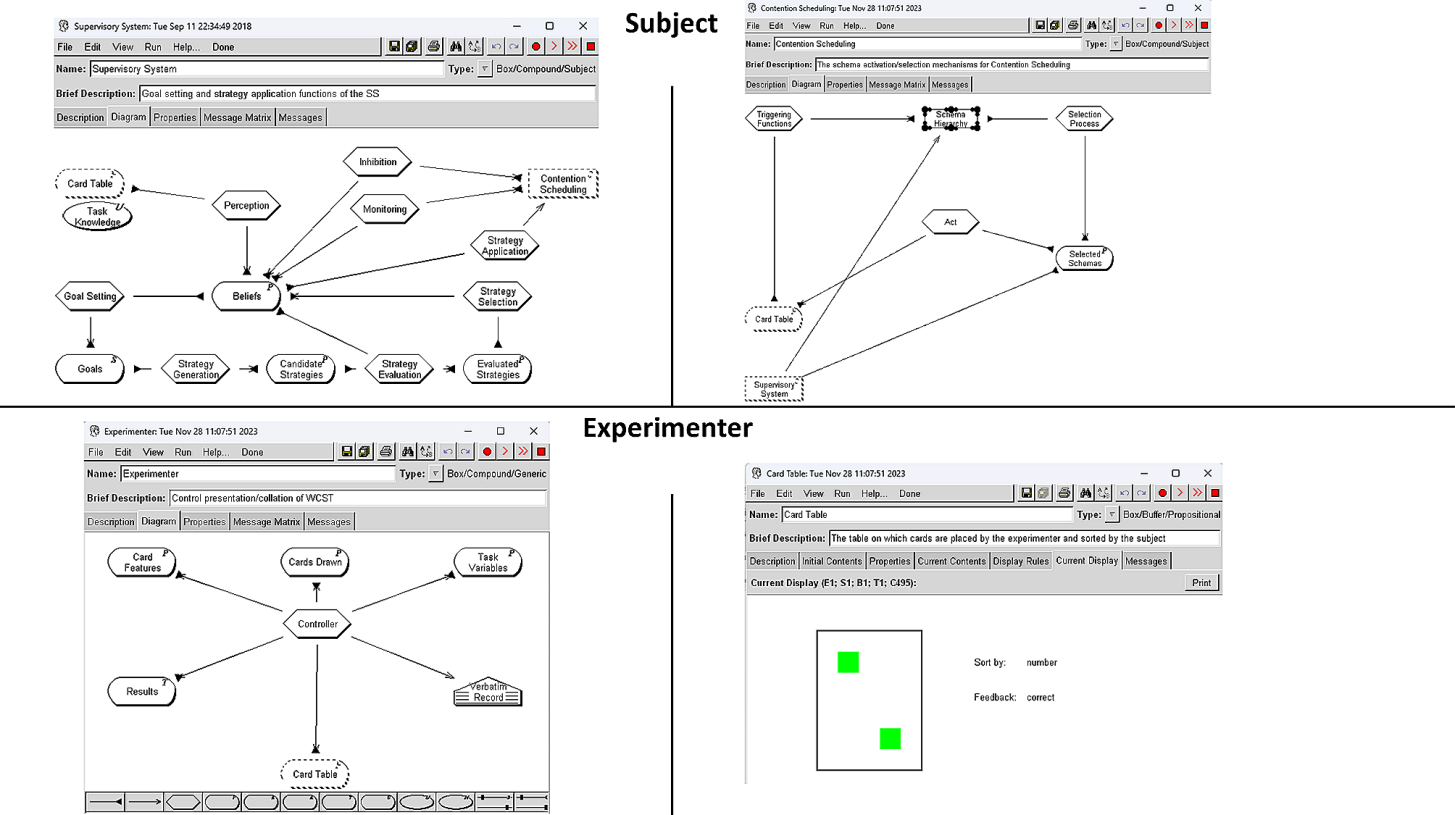
Examples of different sections of the COGENT WCST models as displayed in the software across both Subject and Experimenter

The COGENT models of the WCST were developed based on the specifications of Cooper [48] with slight modifications to facilitate ease of manipulation of the cognitive processes (see Haywood & Baughman [14]). While extensive detail regarding the base model development and underlying theoretical basis are beyond the scope of this paper. The models were developed as per Cooper [48] and based on The Domino Model [50] combined with The Theory of Willed and Automatic Action [51]. Please see Cooper [48] for additional details. Figure 1 presents examples of different sections of the COGENT WCST models as displayed in the software across both Subject and Experimenter.

The ‘subject’ displays the modelled processes, buffers, and networks which facilitate the completion of the WCST. Each process, buffer, and network contains rules and other code which allow for the functioning of the processes and components in line with cognitive theory [48]. The ‘experimenter’ displays the stores, including task rules and procedures, task cards, the task table and the storage of results.

### Model manipulations

Manipulations of the cognitive ability of the COGENT models focused on the three Miyake et al. [43] components of executive functioning; *updating, shifting*, and *inhibition*. Each executive functioning process was directly manipulated within the models to produce greater or lesser impairment. *Updating* and *inhibition* were manipulated to four different levels of functioning (1) Very poor, (2) Poor, (3) Medium and (4) High, while *shifting*, due to model constraints, was manipulated to three levels of functioning, (1) Poor, (2) Medium, and (3) High. All manipulations reflected a percentage of functioning of the Expert Model (the base model that produced no PE). The ‘very poor’ level reflected 20% of the functioning of the expert model, while ‘poor’ reflected 33%, ‘medium’ reflected 66% and ‘high’ reflected 99% of expert model functioning on the particular component. To reflect the cognitive variation seen in humans we developed a model for each combination of the levels of functioning between the three executive functioning components, resulting in 48 unique WCST COGENT models. Once the 48 models were developed, each model was further manipulated to allow for a different number of processing cycles before sorting each card in the WCST. Processing cycles refer to the number of permitted information processing cycles around the entire cognitive system before the model is required to take action (i.e., sort a card in the WCST) [48]. Specifically, each model had a (1) 20-cycle, (2) 40-cycle, and (3) 60-cycle version, this manipulation reflected allowing the models additional processing time before making decisions. This ultimately resulted in 144 unique COGENT WCST models utilised in this research. See Supplementary Materials for an illustration of which code was manipulated.

Values used for each level of functioning on the executive functioning components are provided in Table 1. *Updating* was manipulated within the ‘Monitoring’ process, *Shifting* was manipulated within the ‘Strategy Evaluation’ process, *Inhibition* was manipulated within the ‘Inhibition’ process, and Cycles were manipulated within the ‘Controller’ (see Fig. 1). For additional details regarding manipulations and functioning of the code and processes, refer to Haywood and Baughman [14].

**Table 1:** Values implemented within each executive functioning process in COGENT

Executive Functioning Ability Level	Updating	Shifting	Inhibition
Very poor	0.07	-	-0.10
Poor	0.12	0, 0	-0.22
Medium	0.24	1, -1	-0.44
High	0.37	2, -2	-0.65

Note: Within COGENT, the process of shifting relied on ranking candidate strategies, using whole numbers, at a given time. Thus, manipulations to shifting involved assigning values of ‘0’, representing no shifting, ‘1’ representing moderate shifting towards a strategy and ‘-1’ away from a given strategy, values of ‘2’ represented a strong shift towards a strategy, and ‘-2’ a strong shift away from a strategy. Therefore, Shifting was limited to three levels of functioning

### Participants

A ‘virtual participant’ refers to a computational model completing a single trial of the WCST, mirroring a human participant completing a single trial of the task. Previous piloting of the models revealed approximately 20 virtual participants for each model were required to provide an approximate normal distribution of PE. In line with previous investigations, 20 virtual participants populated each unique model, resulting in a total of 2,880 virtual participants.

### Analysis

A one-way ANOVA and an ANCOVA (controlling for categories obtained and non-perseveration errors) was used to compare the performance of PE on the WCST between the (1) 20-cycle, (2) 40-cycle, and (3) 60-cycle models. One-way analyses were chosen as the groups were determined to be independent. This is because, even though models with the same cognitive profiles were used for the differing permitted number of cycles, the models were not permitted to learn from previous trials. Therefore, each condition (i.e., number of cycles) consisted of independent individual virtual participants who were permitted a differing number of processing cycles. We also compared the number of PE provided by each of the 144 COGENT models to the PE produced by cancer survivors in previously published research [36]. For a model to be classified as a simulation of cancer survivors, they were required to produce a mean number of PE within +/- 1 standard error of the mean PE produced by that group. The comparison WCST data for cancer survivors was taken from Nguyen et al. [36]. This data was used as comparison data as raw PE scores were provided, and the full 128-card WCST version was used. Nguyen et al. [36] administered the WCST to breast cancer survivors all of whom were women over 65 years of age who were at least 50 years old at the time of diagnosis, and who were at least 10 years post-treatment. The cancer survivors either had had chemotherapy (*n* = 27) or local therapies (radiation or surgery; *n* = 30). Table 2 presents the two groups’ demographics and mean PE produced on the WCST.

**Table 2:** Cancer survivor comparison data; demographics and mean PE

Group	Age (SD)	Education (Years)	Perseveration Errors (SD)
Chemotherapy (*n* = 27)			
	72.0 (4.9)	14.6 (2.8)	12.5 (6.9), standard error = 1.33
Local Therapy (*n* = 30)			
	76.7 (5.4)	14.2 (2.1)	12.8 (9.3), standard error = 1.70

Note. SD = Standard Deviation

## Results

### Hypothesis testing

Descriptive performance data for the 20-cycle, 40-cycle, and 60-cycle WCST models is presented in Table 3. Raw scores indicated additional cycles facilitated better performance (i.e., fewer numbers of PE performed).

**Table 3:** Descriptive performance data

	*N*	Mean PE	Std. Deviation	Std. Error	95% Confidence Interval for Mean		Minimum	Maximum
					Lower Bound	Upper Bound		
20-cycles	960	13.55	9.34	0.30139	12.96	14.14	0.00	62
40-cycles	960	6.90	6.31	0.20371	6.50	7.30	0.00	39
60-cycles	960	5.28	5.98	0.19295	4.90	5.65	0.00	39
Total	2880	8.58	8.19	0.15257	8.28	8.88	0.00	62

Note: PE = Perseveration Errors Std. = Standard

A one-way analysis of variance (ANOVA) was used to test hypothesis H1a, that WCST models that allowed for additional card sorting time would produce significantly fewer PE. Specifically, it was anticipated the WCST with 60 cycles would perform significantly fewer PE when compared to WCST models with 40 and 20 cycles, and models with 40 cycles to perform a significantly fewer number of PE than models with 20 cycles. The ANOVA was significant *F*(2, 2877) = 340.01, *p* < .001, η^*2*^ = 0.191, with large-effect indicating a significant main effect of cycle-group on PE performance. The results of the pairwise comparisons following the one-way ANOVA are presented in Table 4.

**Table 4:** H1a pairwise comparisons

Cycles		Mean PE Difference	Std. Error	95%CI	*P* ^a^	d
20-cycles	40-cycles	6.65	0.336	[5.84, 7.45]	< 0.001	0.834
	60-cycles	8.28	0.336	[7.50, 9.08]	< 0.001	1.05
40-cycles	60 cycles	1.63	0.336	[0.823, 2.43]	< 0.001	0.264

Note: PE = Perseveration Errors Std. = Standard. 95%CI = 95% Confidence Interval

*p* = Probability. *t* = *t*-statistic. *d* = Cohens’ *d*

^a^ Bonferroni adjusted for multiple comparisons, significant at 0.05

Supporting hypothesis H1a, the collection of models with additional processing cycles before sorting each card in the task produced significantly fewer PE. There was a large effect between 20 and 40-cycle, and 20 and 60-cycle models, while there was a small effect between 40 and 60-cycle models. This may suggest that while additional cycles may mitigate the performance of PE, there may be diminishing performance returns the larger the number of processing cycles provided.

Next, an analysis of covariance (ANOVA) was used to test hypothesis H1b, that after accounting for number of non-perseveration errors and categories obtained this significant difference would be maintained. In line with H1a and supporting H1b the ANCOVA was significant *F*(4, 2875) = 271.23, *p* < .001, η^*2*^ = 0.274, with large-effect indicating a significant main effect of cycle-group on PE performance after controlling for categories obtained and non-perseveration errors. The results of the pairwise comparisons following the ANCOVA are presented in Table 5.

**Table 5:** H1b pairwise comparisons

Cycles		Mean PE Difference	Std. Error	95%CI	*P* ^a^	d
20-cycles	40-cycles	6.35	0.319	[5.59, 7.11]	< 0.001	0.909
	60-cycles	7.85	0.320	[7.08, 8.61]	< 0.001	1.12
40-cycles	60 cycles	1.50	0.319	[0.731, 2.26]	< 0.001	0.214

Note. Based on estimated marginal means. PE = Perseveration Errors Std. = Standard. 95%CI = 95% Confidence Interval. *p* = Probability. *t* = *t*-statistic. *d* = Cohens’ *d*

^a^ Bonferroni adjusted for multiple comparisons, significant at 0.05

Supporting H1b the collection of models with additional processing time before sorting each card in the task produced significantly fewer PE, after controlling for categories obtained and non-perseveration errors. Once again, there was a large effect between 20 and 40-cycle, and 20 and 60-cycle models, while there was a small effect between 40 and 60-cycle models.

In line with previous WCST computational modelling research [14] the manipulations to the executive functioning components updating, shifting and inhibition nor processing cycles did not notably impact the number of categories obtained or non-perseveration errors. Only two virtual participants (out of 2,880) within the most impaired and fewest cycle model variant (20-cycle very poor updating, poor shifting, very poor inhibition) performed less than the maximum 60 correct sorts and 6 categories obtained within the 128-card trials out of all 144 unique model variants (see Table 6; Participant #14; 58 correct sorts, 5 categories obtained. Participant #20; 56 correct sorts, 5 categories obtained). While an exploratory one-way ANOVA revealed a significant difference between the cycle-groups on non-perseveration errors (NPE) (*F*(2, 2877) = 6.95, *p* < .001, η^*2*^ = 0.011), but this effect was small and reflected a difference of 0.178 NPE between the 20 and 60 cycle group (*p* < .001, Bonferroni corrected). No significant difference in number of NPE performance was found between the 20 and 40 cycle-group (*p* = .077, Bonferroni corrected) nor the 40 and 60-cycle groups (*p* = .077, Bonferroni corrected).

### Determining simulation models

The executive functioning detail of each model variant (i.e., levels of shifting, updating, and inhibition) and the mean number of PE (with standard deviation) performed by each model is shown in Table 6. The models are arranged in ascending order based on their EF ability, beginning with those featuring at least one very poor ability component (models 1–21) and concluding with the model exhibiting the greatest overall EF ability (model 48). For each model executive functioning variant, we provide the results of that model with 20, 40, and 60 processing cycles. We also provide each model’s simulation group, if the number of PE performed by the model falls within +/- one standard error of the mean PE performed by cancer survivors exposed to local therapies (LT; PE = 12.50–15.90), or chemotherapy (CT; PE = 13.27–15.93) [36], they were termed a simulation model of that group. If a model produced more PE than one standard error of the CT group this was denoted as CT+. If a model performed fewer PE than one standard error of the LT group, this was donated as LT-. Cancer survivor comparison data derived from Nguyen et al. [36] is provided in Table 2 (above).

**Table 6:** Performance of PE on the WCST by models with unique EF profiles and processing cycles

Model Number	Model Variant	20-Cycle Mean PE (SD)	Group Simulated	40-Cycle Mean PE (SD)	Group Simulated	60-Cycle Mean PE (SD)	Group Simulated
	(Updating/Shifting/Inhibition)						
1	Poor+/Poor/Poor+	43.10 (9.03)	CT+	24.40 (7.45)	CT+	17.05 (5.20)	CT+
2	Poor/Poor/Poor+	25.65 (6.05)	CT+	16.85 (6.86)	CT+	15.20 (5.06)	LT, CT
3	Medium/Poor/Poor+	25.80 (7.92)	CT+	14.80 (5.46)	LT, CT	16.10 (7.20)	CT+
4	High/Poor/Poor+	28.05 (7.06)	CT+	14.75 (7.16)	LT, CT	15.85 (5.37)	LT, CT
5	Poor+/Medium/Poor+	25.15 (3.53)	CT+	11.50 (2.54)	LT-	10.20 (1.32)	LT-
6	Poor+/Medium/Poor	15.65 (2.48)	LT, CT	7.00 (1.30)	LT-	3.90 (1.33)	LT-
7	Poor+/Medium/Medium	10.00 (2.10)	LT-	4.00 (1.59)	LT-	1.95 (1.67)	LT-
8	Poor+/Medium/High	6.80 (2.50)	LT-	2.45 (1.50)	LT-	2.00 (1.23)	LT-
9	Poor+/High/Poor+	23.10 (3.28)	CT+	11.60 (2.33)	LT-	10.80 (1.64)	LT-
10	Poor+/High/Poor	15.60 (5.47)	LT, CT	6.65 (1.98)	LT-	3.10 (1.68)	LT-
11	Poor+/High/Medium	9.60 (1.88)	LT-	3.20 (1.61)	LT-	2.45 (1.39)	LT-
12	Poor+/High/High	7.35 (1.69)	LT-	2.45 (1.82)	LT-	2.00 (1.65)	LT-
13	Poor+/Poor/Poor	17.65 (4.82)	CT+	10.75 (5.65)	LT-	8.65 (4.40)	LT-
14	Poor+/Poor/Medium	15.50 (5.86)	LT, CT	6.80 (5.21)	LT-	5.80 (4.09)	LT-
15	Poor+/Poor/High	8.65 (2.83)	LT-	7.35 (5.88)	LT-	8.20 (8.48)	LT-
16	Poor/Medium/Poor+	28.30 (6.89)	CT+	11.15 (1.63)	LT-	9.80 (2.07)	LT-
17	Medium/Medium/Poor+	21.60 (3.60)	CT+	9.80 (1.54)	LT-	10.50 (1.91)	LT-
18	High/Medium/Poor+	20.40 (2.56)	CT+	9.75 (1.68)	LT-	10.15 (1.84)	LT-
19	Poor/High/Poor+	22.40 (3.17)	CT+	10.75 (2.07)	LT-	9.70 (1.53)	LT-
20	Medium/High/Poor+	22.40 (3.02)	CT+	10.35 (1.38)	LT-	10.45 (1.19)	LT-
21	High/High/Poor+	22.30 (2.92)	CT+	10.90 (1.62)	LT-	9.80 (2.02)	LT-
22	Poor/Poor/Poor	15.90 (6.72)	LT, CT	8.85 (4.33)	LT-	7.10 (4.93)	LT-
23	Poor/Poor/Medium	12.45 (4.83)	LT-	8.50 (5.74)	LT-	4.55 (2.93)	LT-
24	Poor/Poor/High	10.05 (4.65)	LT-	6.25 (5.10)	LT-	6.70 (4.61)	LT-
25	Poor/Medium/Poor	11.15 (1.76)	LT-	7.50 (1.19)	LT-	1.45 (0.999)	LT-
26	Poor/Medium/Medium	7.55 (1.50)	LT-	0.900 (0.788)	LT-	0.050 (0.224)	LT-
27	Poor/Medium/High	4.60 (2.06)	LT-	0.450 (0.605)	LT-	0 (0)	LT-
28	Poor/High/Poor	12.15 (1.57)	LT-	7.45 (0.999)	LT-	1.60 (0.940)	LT-
29	Poor/High/Medium	7.75 (1.25)	LT-	0.900 (1.29)	LT-	0.050 (0.223)	LT-
30	Poor/High/High	4.90 (1.74)	LT-	0.350 (0.489)	LT-	0 (0)	LT-
31	Medium/Poor/Poor	16.05 (6.92)	CT+	8.80 (4.37)	LT-	6.45 (6.24)	LT-
32	Medium/Poor/Medium	10.80 (5.60)	LT-	6.89 (5.89)	LT-	6.15 (3.60)	LT-
33	Medium/Poor/High	5.95 (3.82)	LT-	5.90 (3.67)	LT-	7.45 (9.13)	LT-
34	Medium/Medium/Poor	9.15 (1.90)	LT-	7.80 (1.51)	LT-	0.100 (0.308)	LT-
35	Medium/Medium/Medium	6.60 (0.883)	LT-	0.100 (0.308)	LT-	0 (0)	LT-
36	Medium/Medium/High	2.40 (1.35)	LT-	0 (0)	LT-	0 (0)	LT-
37	Medium/High/Poor	8.95 (2.04)	LT-	7.45 (1.32)	LT-	0.100 (0.308)	LT-
38	Medium/High/Medium	7.55 (1.19)	LT-	0.150 (0.366)	LT-	0 (0)	LT-
39	Medium/High/High	1.95 (1.23)	LT-	0 (0)	LT-	0 (0)	LT-
40	High/Poor/Poor	16.60 (4.78)	CT+	9.05 (7.70)	LT-	6.35 (4.22)	LT-
41	High/Poor/Medium	11.05 (5.12)	LT-	8.35 (7.50)	LT-	6.55 (4.26)	LT-
42	High/Poor/High	7.90 (4.05)	LT-	10.35 (7.12)	LT-	7.95 (5.11)	LT-
43	High/Medium/Poor	16.10 (4.31)	CT+	9.85 (3.08)	LT-	6.75 (4.14)	LT-
44	High/Medium/Medium	7.60 (1.76)	LT-	0.400 (0.503)	LT-	0.200 (0.616)	LT-
45	High/Medium/High	4.35 (1.78)	LT-	0.550 (0.605)	LT-	0 (0)	LT-
46	High/High/Poor	8.10 (1.59)	LT-	7.35 (1.14)	LT-	0 (0)	LT-
47	High/High/Medium	6.50 (0.889)	LT-	0 (0)	LT-	0 (0)	LT-
48	High/High/High	1.30 (1.30)	LT-	0 (0)	LT-	0.050 (0.223)	LT-

Note: Group Simulated shows the fit of each model to corresponding target group (LT = Local Therapy, CT = Chemotherapy), +/- denotes performance levels above or under boundary ranges. PE = Perseveration Errors. *SD* = Standard Deviation

**Fig. 2:**
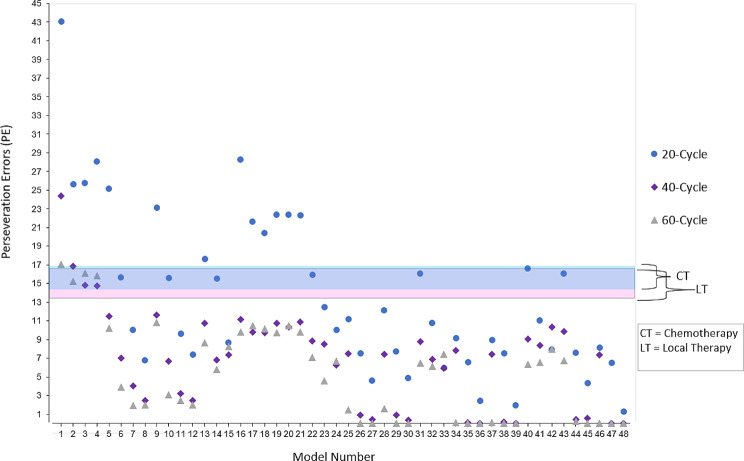
COGENT models’ mean PE performance overlaid with the chosen cancer survivors simulation boundary [36]

The models PE performance (derived from Table 6) overlaid with the chosen +/- one standard error simulation boundary from the mean PE performed by cancer survivors [36] are provided in Fig. 2, demonstrating the high amount of variability in the mean number of PE performed by the models (lowest mean PE = 0, highest mean PE = 43.10).

As expected by allowing additional card sorting time, the computational models that had significant cognitive impairments and that produced more PE than the target simulation groups, performed a reduced number of PE that simulated the performance of cancer survivors. Eight unique models simulated the number of PE provided by both groups of cancer survivors (chemotherapy naïve and chemotherapy treated) on the WCST. Four 20-cycle models (model numbers 6, 10, 14 and 22), two 40-cycle models (model numbers 3 and 4), and two 60-cycle models (model numbers 2 and 4) simulated the group’s performance, with only a single model (model number 4) simulated the cancer survivors’ performance across more than one processing cycle group (20 and 60-cycle). Three out of the four 20-cycle simulation models had very poor updating (model numbers 6, 10 and 14), all of the 40-cycle (model numbers 3 and 4), and 60-cycle (model numbers 2 and 4) simulation models had very poor inhibition. All 20-cycle model variants that had very poor inhibition exceeded simulation bounds by producing too many PE. This may suggest that the additional processing cycles were able to mitigate the effects of very poor inhibition for some model variants. Further supporting this suggestion, while all the 20-cycle models that had very poor inhibition produced too many PE, the majority of 40-cycle and 60-cycle models that had very poor inhibition provided too few PE to be simulation models (model numbers 5, 9, 16–21). In fact, only two 40-cycle very poor inhibition models (model number 1 and 2) exceeded simulation bounds and only a single 60-cycle very poor inhibition model (model number 1) exceeded the boundary. In addition to very poor inhibition four of the 40-cycle and 60-cycle simulation models had poor shifting, although while 40 and 60-cycle models simulated model number model number 4 (with medium shifting), only the 60-cycle model was able to simulate model 2, which had poor shifting ability.

Overall, it seems that deficits to updating is characteristic of 20-cycle models that simulated the cancer survivors’ performance of PE, but additional processing cycles (40 and 60 cycles) could mitigate the effects of substantial deficits to inhibition, and ultimately explain the lack of differences in PE often observed been cancer survivors and control groups. Further, it seems that longer processing time (i.e., additional cycles) may not only mitigate the effects of singular cognitive process deficits, but also the interactive and combined effects of specific deficits to updating, shifting, and inhibition processes.

## Discussion

This study aimed to (1) examine if allowing the computational models additional card sorting time could compensate for cognitive impairments, as evidenced by the number of PE on the WCST; and (2) explore the profiles of cognitive impairments and the amount of allowable card sorting time of the computational models that simulate the number of PE performed by cancer survivors on the WCST. The computational approach used allowed the isolation of models representing cancer survivors with specific profiles of cognitive ability who may benefit in their daily lives, if given additional time to think and reason. By directly impairing the computational cognitive system through precise manipulations we were able to directly observe the *causal* influence of manipulation to EF processes and allowable time on performance on the WCST. The computational models used were also broad and varied accounting for cognitive heterogeneity as well as for the interactions between cognitive processes of differing abilities [14, 52–54]. Ultimately, the hypothesis that computational models allowing for additional processing time would produce significantly fewer PE was supported. Further, eight unique computational models accurately simulated the performance of cancer survivors.

Cognitive slowing is a key factor of CRCI [3, 11] and recent qualitative evidence suggests that impairments to speed of information processing can have profound impacts on cancer survivors daily activities, relationships, occupational functioning, distress, and social functioning [4, 5]. Our research supports, but extends upon, Kesler et al. [12]’s proposal that allowing additional time for information processing and decision-making may be a useful strategy for some cancer survivors with CRCI. A large improvement in WCST performance between the 20 and 40-cycle models, with significant, but notably less improvement between 40 and 60-cycle models was observed. This may suggest that while allowing for additional processing and decision-making time can have positive effects, these may be diminishing beyond a certain magnitude. There is potential for a particular cut-off point or threshold whereby additional time may not improve performance, however, incremental improvements in WCST performance were observed across our three processing cycle groups. Our results do however suggest that the amount of additional processing time needed to mitigate the effects of CRCI may depend on a cancer survivors’ particular profile of cognitive ability with more severe impairments, particularly to inhibition, requiring significantly additional processing time.

Additional processing cycles (40 and 60) were also able to largely mitigate the negative effects of impairments to inhibition on the WCST, and this may largely account for the often similar production of PE on the WCST between cancer survivors and controls [12, 36]. Mechanistically, this is sensical as the function of the inhibition cognitive process is to inhibit a predominate response to allow for a more appropriate response to the performed strategy [43]. Allocating additional time (i.e., processing cycles) may alleviate the effects of inhibition impairments by providing cancer survivors with the necessary time for other cognitive processes, such as updating working memory and shifting mental set, to more prominently influence decision-making (e.g., selecting a sorting strategy), thus reducing reliance on the inhibition process to counteract initiation of the preceding card sorting strategy (e.g., the predominant response). Additionally, more processing cycles may partly mitigate ‘cognitive slowing’ of the inhibition process [3, 11]. Providing the inhibition process with extra time may enable it to achieve a performance level similar to that without impairment (i.e., without cognitive slowing) under more time-restricted conditions. To mitigate the effects of more severe global executive functioning impairments (across updating, shifting and inhibition), our results suggest that only a significant amount of additional permitted time (i.e., the 60-cycle models) was required. However, for the most severe global executive functioning deficits, even three-fold additional processing cycles (as in the 60-cycle models) proved inadequate to mitigate the effects and replicate the performance of cancer survivors. This finding may support Kesler et al. ’s [12] claim that for some cancer survivors, due to their cognitive profile, allowing additional time may not be an effective strategy to mitigate the effects of CRCI.

Practically, our findings suggest that, in collaboration with the emerging literature, for some cancer survivors with CRCI, allowing additional time may result in similar cognitive performance accuracy when compared to cancer and treatment naïve controls. Given the impact of CRCI on various life domains implementing an increased time strategy may have the potential to mitigate negative outcomes. Notably, CRCI is a key factor influencing a cancer survivors’ ability to work [55–57], and this strategy may have particular utility in facilitating return-to-work for cancer survivors with CRCI and mitigation of the potential for poorer occupational performance [4, 56–58]. For instance, in occupational settings, human resource officers, managers, and co-workers could strategically manage workload and deadlines to accommodate additional processing time. its possible that additional processing time may result in occupational output of similar quality as prior to the cancer survivor experiencing CRCI. Our results suggest that the efficacy of this strategy, and amount of additional time required, may differ depending on the cancer survivor’s specific cognitive profile and the cognitive demands of their work [4, 5]. In light of the recent emphasis on enhancing occupational outcomes for cancer survivors with CRCI [59] and the understanding that CRCI is a key factor regarding work ability [55], future research both empirically and computationally, is required to better understand the nuance between CRCI-profiles, permitted time, and occupational outcomes.

### Study limitations

This research had a number of limitations. We only manipulated the components of Miyake and colleagues EF processes [43]. While these are key cognitive components impacted by CRCI [3], lower-level processes, such as working memory capacity and speed of information processing may be useful to include in model variants and explain performance [60]. We also implemented executive function manipulations at only three or four levels of ability and for processing cycles of only three different levels. While this provided extensive variance as well as unique cognitive profiles and experimental groups [61], future work examining greater variability would strengthen this research. We also used data from a single study, which exclusively included mid-to-older adults, to explore models that simulated the performance of cancer survivors. It is important to note that older age is associated with slowed speed of information processing [54], providing a potential confounding variable in understanding the applicability of the results. Therefore, the simulation findings are only applicable to that particular study sample’s characteristics, including cancer types and stages, treatment types, time since cancer and treatments, comorbidities, and demographic factors. Additionally, while the manipulation of processing cycles provides a good representation of permitted time, we were unable to represent cycles in units of time limiting our ability to provide detail of the processing time requirements in units of time. We also only examined in depth the PE metric of the WCST. While this metric offers perhaps the most ecological validity regarding outcome prediction [14], the other metrics of the WCST still offer utility. Further research should extend this work by including the other WCST metrics. Lastly, our research only focused on allowable task completion time, other cognitive compensation strategies, such as self-pacing [60], have been developed to improve performance on the WCST and may be used complementary. Future research should look to extend upon this research and mitigate these limitations through the use of other modelling approaches, such as dynamical systems [62] and other forms of artificial neural network models [39, 63], as well as corroborate computational findings with human-based research.

## Conclusion

Computational evidence is provided that allowing additional processing time may be a useful strategy for some cancer survivors with CRCI. Allowing additional processing time may mitigate the effects of some impaired cognitive profiles and reduce the number of perseveration errors produced on the WCST. A moderate number of additional processing cycles seemed to provide its positive influence on performance primarily through mitigating the impacts of severe inhibition impairments, however for more severe global executive function impairments substantial additional processing cycles were required. For the most severe global cognitive deficit models, the additional processing cycles were unable to improve performance equivalent to the average performance of cancer survivors. Our findings have implications for the development of practical strategies, such as workload and deadline management in occupational settings, which may mitigate the negative effects of CRCI. Future computational and empirical research should seek to further elucidate the nuances of processing time and cognitive performance for cancer survivors with CRCI.

### Electronic supplementary material

Below is the link to the electronic supplementary material.

Supplementary Material 1

## Data Availability

The data may be supplied at by requesting access via the corresponding author.

## Abbreviations

CRCI: Cancer related cognitive impairment
WCST: Wisconsin Card Sorting Task
PE: Perseveration Errors
Std.: Standard
95%CI: 95% Confidence Interval
p: Probability
t: t-statistic
d: Cohens’ d
