# Supplementary material for: It’s about time: mitigating cancer-related cognitive impairments through findings from computational models of the Wisconsin Card Sorting Task
Source: BMC Cancer. 2024 Jul 4;24:798. doi: 10.1186/s12885-024-12545-7 (PMC11223404; doi:10.1186/s12885-024-12545-7)
Supplement: Supplementary file 1 — Supplementary Material 1 [file 12885_2024_12545_MOESM1_ESM.docx]

**It's About Time: Mitigating Cancer-Related Cognitive Impairments Through Findings from Computational Models of the Wisconsin Card Sorting Task**

*Supplementary Materials*

Supplementary figure 1 below shows the code which was manipulated within the COGENT models for each executive functioning component, as well as the number of cycles to deal with feedback.


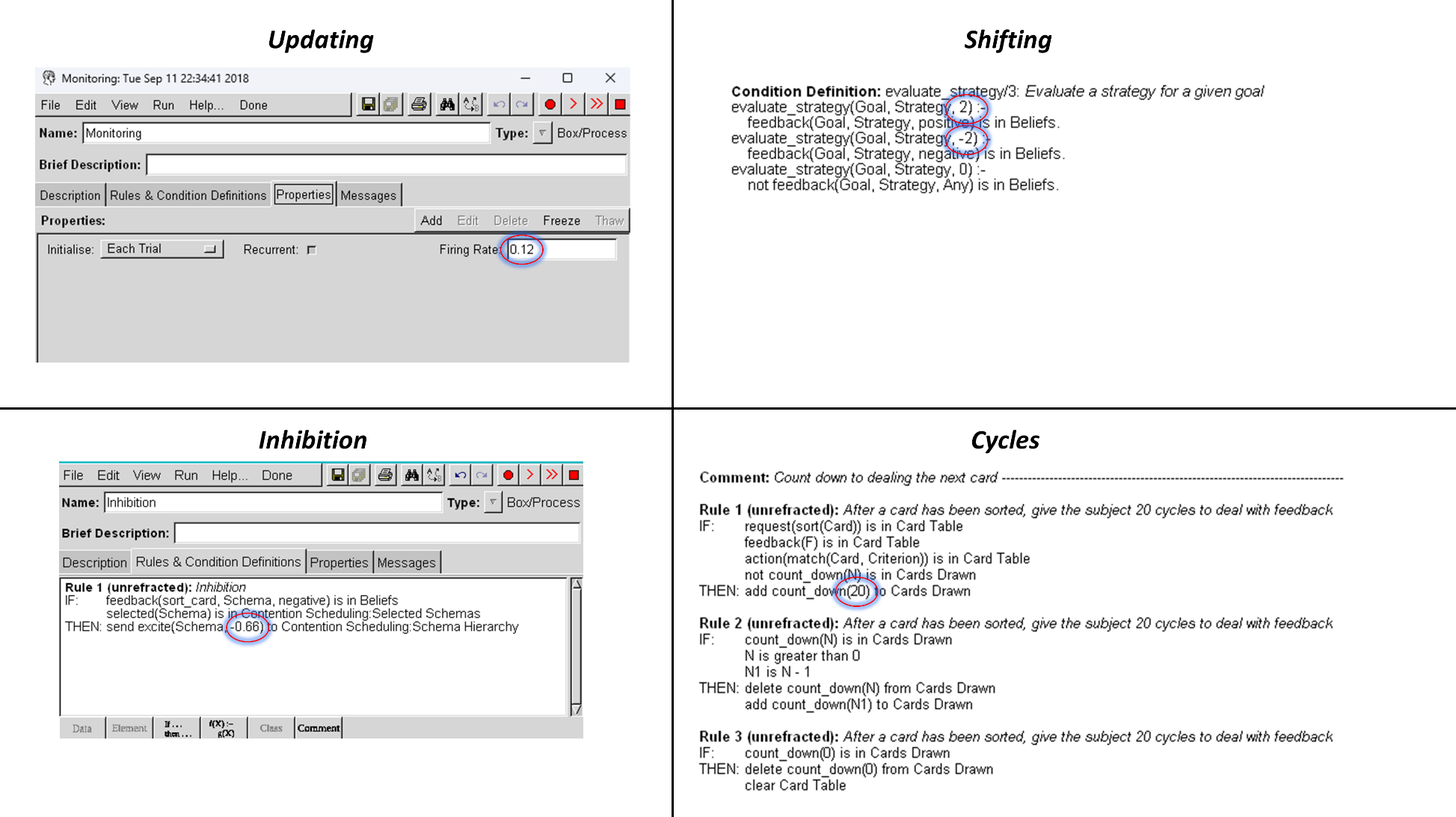


***Supplementary Figure 1.*** Code manipulations to influence executive functioning component ability.
